# Protein-mediated interactions in the dynamic regulation of acute inflammation

**DOI:** 10.32604/biocell.2023.027838

**Published:** 2023-05-19

**Authors:** RYAN STARK

**Affiliations:** Department of Pediatric Critical Care Medicine, Vanderbilt University Medical Center, 2200 Children’s Way, 5121 Doctors’ Office Tower, Nashville, TN 37232-9075

**Keywords:** Protein Interactions, Inflammation, Sepsis, RNA, DNA, Therapeutics

## Abstract

Protein-mediated interactions are the fundamental mechanism through which cells regulate health and disease. These interactions require physical contact between proteins and their respective targets of interest. These targets include not only other proteins but also nucleic acids and other important molecules as well. These proteins are often involved in multibody complexes that work dynamically to regulate cellular health and function. Various techniques have been adapted to study these important interactions, such as affinity-based assays, mass spectrometry, and fluorescent detection. The application of these techniques has led to a greater understanding of how protein interactions are responsible for both the instigation and resolution of acute inflammatory diseases. These pursuits aim to provide opportunities to target specific protein interactions to alleviate acute inflammation.

## Introduction

**FIGURE 1:**
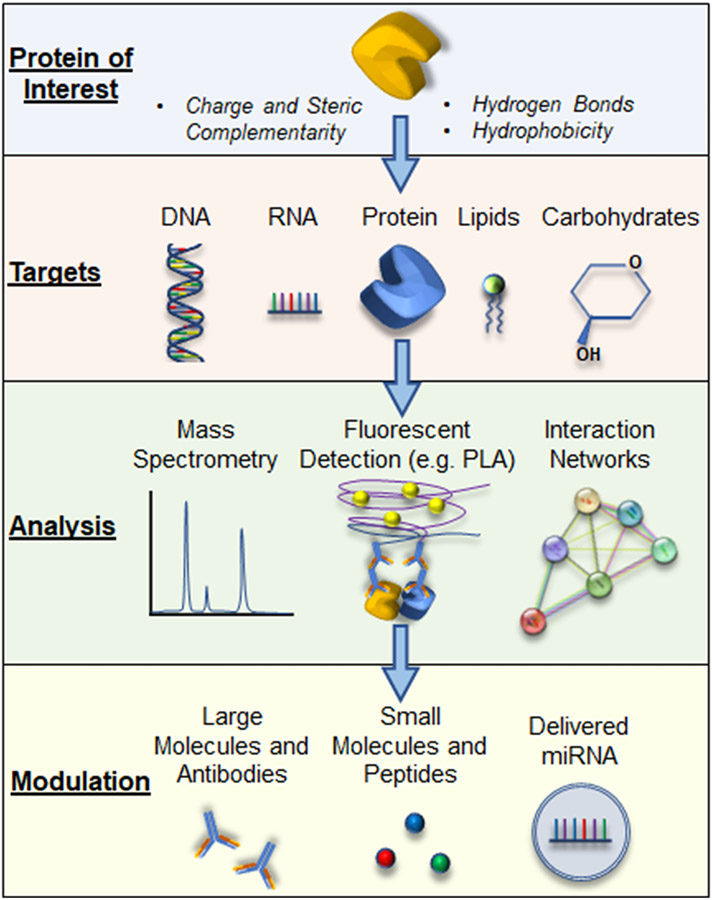
A schematic representation outlining how proteins of interest interact with possible binding partners and the potential therapeutic avenues that can be employed to modulate the known interactions.

The regulation of cellular processes by proteins forms the foundation of all cellular biology and pathobiology. Fixed or transient interactions of proteins with their respective targets of interest allow for broad, dynamic modifications of cell mechanisms for both health and disease (De Las Rivas and Fontanillo, 2010). Targets of these interactions include other proteins, RNA, DNA, carbohydrates, and lipids. These interactions, in turn, regulate enzymatic alterations, molecular transport, cell expression patterns, and global signal transduction. While proper protein-mediated interactions are necessary for cell homeostasis, the importance of these interactions is no less true in acute inflammatory conditions. Under these settings, the dynamic protein-mediated interactions provide the dual purpose of initiating an appropriate response to the inflammatory challenge but also a mechanism to temper the response when the threat is abated. These opposing responses ideally work in concert to control the response and restore homeostasis, with the survival of the host as the outcome. However, dysregulation of these responses can lead to enhanced or perturbed protein-mediated interactions, causing the host to succumb to the disease. This mini-review will provide broad strokes on the mechanisms of protein-mediated interactions with a variety of cellular components, how alterations in these interactions result from and produce disease in acute inflammatory conditions, and suggest potential therapeutic avenues to regulate these mechanisms to tamper disease states (Fig. 1).

## Mechanisms of Protein-Mediated Interactions

### Protein-protein interactions

In the broadest sense, protein-protein interactions form the mechanisms through which multi-protein complexes are created (Dinant et al., 2009). The interactions fundamentally depend on the amino acid sequences and their associated charge of the proteins forming the interaction. In some instances, gaps are formed in protein folding that allows for other molecules or proteins to fill. This process is called steric complementarity and forms the “lock-and-key” approach to protein binding (Blaney et al., 1982). Comparably, electrostatic charges of amino acid sequences form the basis for protein folding (Roca et al., 2007). When basic residues are in proximity to acidic residues, the electrostatic charge pulls the opposingly charged sequences together and can be used to form interactions between adjacent proteins. These interactions and steric relationships, create unique docking sites for protein-protein binding (Vakser, 2014). A vast number of these sequences, called conserved domains, have been cataloged and can be readily assessed (https://www.ncbi.nlm.nih.gov/Structure/cdd/cdd.shtml). In addition, both the hydrophobicity and shared bonding of hydrogen account for other mechanisms through which proteins can interact to modify one another (Jiang and Lai, 2002).

### Protein-DNA and protein-RNA interactions

Similar to protein-protein interactions, it has been long recognized that proteins can form direct binding connections with nucleic acid structures (Blake and Oatley, 1977). Protein interactions with strands of various nucleic acid sequences are necessary for DNA-mediated production of proteins. Often these protein-mediated interactions modify various aspects of this process, from localization within the cell to sequence stability and editing (Re et al., 2014). But while the transcription of DNA to RNA and subsequent translation of RNA to amino acids is well understood, it is approximated that up to 80% of human DNA, and likewise RNA, do not code for proteins (Consortium, 2012). Instead, much of this genetic material has regulatory functions that include the modulation of the cellular environment through protein-mediated interactions (Elkon and Agami, 2017). In particular, non-coding RNA (ncRNA) are RNA sequences, either long (>200 bps) or short (<200 bps), that cannot code for a protein. Despite this, ncRNAs have been shown to regulate many aspects of cell biology, including through their regulation of proteins (Kazimierczyk et al., 2020). Understanding the role of these interactions is still in its infancy, but it has been suggested that the more complex a protein network is, the more likely it has interactions with ncRNAs, such as microRNAs (miRNAs), to alter the multi-protein complex function through dedicated ncRNA docking sites (Liang and Li, 2007).

### Other direct protein interactions

Though protein-protein and protein-DNA/RNA interactions are important in signaling, proteins can interact with other cellular components, namely lipids, and carbohydrates. Like the mechanisms previously mentioned, protein-lipid or protein-carbohydrate interactions often depend on charge and hydrophobicity that allow for specific binding. In the case of carbohydrates, the many hydroxyl groups (-OH) present in carbohydrates allow for the potential of hydrogen bond crosslinking (Zhang et al., 2021). Likewise, electrostatic interactions and divalent cationic bridges also account for protein-carbohydrate interactions, similar to other protein-mediated mechanisms mentioned (Boraston et al., 2004). In a similar context, protein-lipid interactions are crucial for supporting cell membrane integrity. Binding sites found on proteins help form these interactions. In particular, pleckstrin homology domains on proteins have been well-established to bind phosphatidylinositol lipids, which aid in cytoskeleton rearrangements (Corey et al., 2020).

### Detection methods

The capture of protein-mediated interactions can be challenging due to their dynamic and transient nature. Typically, for a protein-mediated interaction to be considered meaningful, the interaction should be considered intentional (i.e., not via Brownian motion) and with a specific biological purpose to induce a physiologic change (De Las Rivas and Fontanillo, 2010). Some more basic detection methods include affinity purification-mass spectrometry and co-immunoprecipitation, which identify protein-binding partners (Rao et al., 2014; Cozzolino et al., 2021). X-ray crystallography provides three-dimensional structure and visualization of interactions, making this technique exceptionally important in the lock-and-key design of drug targets. Other fluorescent microscopic techniques, such as proximity ligation assays (PLA) and förster resonance energy transfer (FRET), provide some spatial understanding of protein-mediated interactions (Ivanusic et al., 2014). Assessing the strength of these interactions can be performed with such techniques as atomic force microscopy (AFM) and biolayer interferometry (Whited and Park, 2014; Barrows and Van Dyke, 2022). More recently, computational techniques have been developed to assess the complex and dynamic relationships that protein-mediated interactions have on cell function. These include computational models to predict possible interactions and large-scale databases to test known binding partners or interactions within biological networks (Ding and Kihara, 2018; Droit et al., 2005; Yang et al., 2020). For instance, the STRING Database (https://string-db.org/) allows for the analysis of both physical and functional interactions between multiple proteins of interest. Understanding the mechanisms of protein-mediated interactions, and the tools to assess them, have allowed for investigations into the relevance of these relationships during acute inflammation.

## Protein-Mediated Interactions in Acute Inflammation

At the most basic level, protein-mediated interactions are the drivers of the cellular response to acute inflammatory challenges. Starting with either cytokines, damage-associated or pathogen-associated molecular patterns (DAMPs and PAMPs), interactions of these molecules with pattern recognition receptors (PRRs) start a signaling cascade. During an inflammatory challenge, this cascade is initiated and propagated by a series of protein-mediated processes that include protein-protein, protein-DNA/RNA, protein-carbohydrate, and protein-lipid interactions to drive the biological response (Schweppe et al., 2015). One example is lectins, which are non-enzymatic carbohydrate-binding proteins and important instigators and regulators of the host response to pathogens (Mason and Tarr, 2015). Indeed, C-type lectin receptors (CLRs) are one of the primary classes of PRRs that form a crucial first step to initiating an inflammatory response to pathogenic challenge (Geijtenbeek and Gringhuis, 2009). Another well-described class of PRRs is toll-like receptors (TLRs), which are homo or heterodimeric proteins that recognize foreign or endogenous proteins, lipids, or DNA/RNA to start the inflammatory response (Kawasaki and Kawai, 2014). Further protein-mediated interactions at the cellular surface include the previously mentioned protein-lipid interactions necessary for the movement of proteins on the cell surface and endocytosis-mediated signaling (Ewers and Helenius, 2011).

The protein-mediated signals that occur at the plasma membrane and beyond are typically the result of a series of assembling and dissembling multi-protein complexes that exert their effect on various parts of the cell. These multi-protein complexes aid in signal transduction through a variety of post-translational modifications to induce protein targets to carry out specific functions (Ramazi and Zahiri, 2021). Signal transduction through inflammatory cascades is also under the influence of either internal or external factors, such as non-protein coding RNA products. These products can, in turn, either enhance or abate the inflammatory response. The overall downstream protein-mediated processes related to this are far beyond the scope of this mini-review; however, the focus will be provided on two crucial aspects of inflammatory signaling: protein-RNA interactions in modulating the inflammatory response and protein-protein interactions in inflammation-mediated metabolism.

### NcRNAs in pathogen-mediated inflammation

As mentioned previously, a majority of DNA does not code for a specific protein. Nevertheless, these non-coding regions have important regulatory functions in host defense (Li et al., 2021). In one recent study, in neonates with sepsis, which is a severe immunological response to a pathogen, 89 long non-coding RNAs (lncRNAs) were found to be altered (Bu et al., 2020). One well-described lncRNA is metastasis-associated lung adenocarcinoma transcript 1 (MALAT1). Circulating levels of MALAT1 have been shown in patients with sepsis and coronavirus disease (COVID)-19, and these levels correlate with the severity of the disease (Chen et al., 2020; Huang et al., 2022). MALAT1 is known to interact with several proteins involved in RNA processing and gene transcription; however, a study using a combination of RNA pull-down and mass spectrometry showed that MALAT1 has potentially up to 127 protein binding partners. In particular, MALAT1 binds to a protein called depleted in breast cancer 1 (DBC1) and in doing so, increases a protein called sirtuin 1 (SIRT1) which is a regulator of cellular metabolism (Chen et al., 2017). MALAT1 also functions as a miRNA sponge (Liu et al., 2019). The term “sponge” is used when direct interactions occur between a miRNA and another molecule, which inhibits the miRNA of interest from carrying out its regulatory function (Bak and Mikkelsen, 2014). A meta-analysis of samples from patients with sepsis demonstrated that 39 miRNAs were differentially regulated (Formosa et al., 2022). One of these, miR-125B, has been associated with MALAT1. Data has shown that as MALAT1 expression increased, miR-125B decreased in proportion, and this again correlated with disease severity (Le and Shi, 2022). In this sense, miRNA expression patterns have been suggested as biomarkers to delineate disease and the severity of illness (Dumache et al., 2015). But beyond expression patterns, miRNAs have functional roles mediated through direct interactions. Once produced in the nucleus, they can exert their effect locally, such as via histone modifications or RNA silencing with subsequent protein reduction. They can also be packaged into exosomes for more distal impact. TLR4, a well-studied PRR whose primary agonist is gram-negative lipopolysaccharide, produces an array of miRNAs when activated, but in turn, its signaling cascade is also modulated at various points by miRNAs (Szilagyi et al., 2019). Thus, it can be seen that non-protein coding RNA products are mechanisms through which the host fine-tunes the inflammatory response to a challenge via direct protein-RNA or RNA-RNA interactions.

### Protein interactions in the regulation of inflammatory metabolism

**FIGURE 2:**
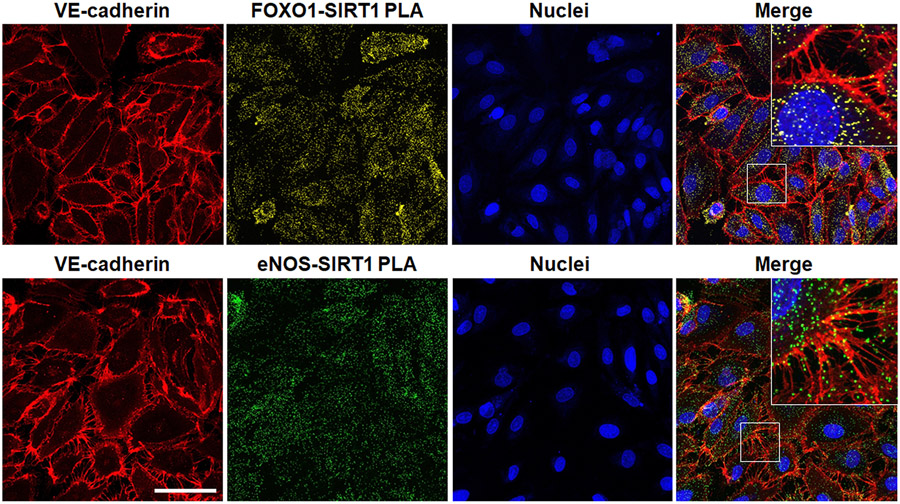
Demonstration of forkhead box 1 (FOXO1, top) or endothelial nitric oxide synthase (eNOS, bottom) and sirtuin 1 (SIRT1) interactions in human microvascular endothelial cells during acute challenge by gram-negative lipopolysaccharide (LPS). Immunofluorescence staining of endothelial VE-cadherin (red) and FOXO1-SIRT1 (yellow) or eNOS-SIRT1 (green) interactions by proximity ligation assay (PLA) with nuclei (blue). Merged images with inserts shown at the far-right demonstrate FOXO1-SIRT1 and eNOS-SIRT1 protein-protein interactions within the projections of VE-cadherin, necessary for maintaining tight gap junctions, between adjacent endothelial cells during pathogenic inflammatory challenge. Captured on a Zeiss LSM 880 inverted confocal microscope. Bar=50 μm. Unpublished image courtesy of R. Stark derived from experiments outlined in Stark et al. (2022).

The crux of a host response to combat a severe inflammatory challenge is to ensure that the energy needs of the cells are met (Lee and Huttemann, 2014). Under resting conditions, much of the energy produced as adenosine triphosphate (ATP) is through mitochondrial oxidative phosphorylation as opposed to glycolysis, the latter of which also generates ATP, though to a much lesser degree (Bonora et al., 2012). However, in times of stress, the phosphate group is quickly utilized, producing adenosine monophosphate and adenosine diphosphate, which must be readily restored to ATP. Though in the resting state, oxidative phosphorylation is the premier source of ATP generation, during an infectious challenge, the path of energy production pivots towards glycolysis (Van Wyngene et al., 2018). This described “metabolic shift” is important in host defense and survival (Cheng et al., 2016). The processes that regulate this shift are highly dependent on protein-mediated interactions and disruptions can lead to an altered immunometabolic phenotype. Hypoxia-inducible factor 1 alpha (HIF-1α), a key regulator of metabolism during stress, is impacted by the capture of a protein called von Hippel–Lindau protein (VHL), leading to HIF-1α degradation (Weidemann and Johnson, 2008). Upregulation of HIF-1α occurs during sepsis, and its destruction by protein-protein interactions with VHL is thought to shift the cells toward fatty-acid metabolism and an anti-inflammation phenotype (Fitzpatrick, 2019). Similarly, SIRTs are another class of proteins that are thought to be important mediators of metabolism during times of acute stress. Of the seven subtypes of SIRTs known, SIRT1 is the best described in its link with metabolism (Chang and Guarente, 2014). SIRT1 has a vast array of proteins it interacts with through its deacetylation activity (McBurney et al., 2013). In endothelial cells, SIRT1 interacts with several key regulatory and homeostatic proteins (Stark et al., 2022). These interactions include not only those with metabolism-related proteins, such as endothelial nitric oxide synthase and forkhead box O1, but also with structural proteins, such as vascular endothelial cadherin (VE-cadherin), putting SIRT1 protein networks at the crossroads of structure and function (Fig. 2). In total, alternations or disruptions in these protein-mediated interactions drive the clinical phenotypes seen and provide opportunities for potential therapeutic intervention.

## Methods of Targeting Protein Interactions

The importance of understanding protein-mediated interactions cannot be understated, especially in the context of drug discovery to mitigate disease. Potentiating or inhibiting key interactions during a disease process provides the possibility to hasten or restore homeostasis. As mentioned previously, the foundation of most protein-mediated interactions involves a lock-and-key interaction. Interactions can be directly targeted through orthosteric mechanisms or via indirect targeting through allosteric mechanisms (Nussinov and Tsai, 2012). Lu et al. (2020) have already provided an excellent and thorough review of this subject matter, so this discussion will focus on key highlights and generalized methods of targeting protein interactions, especially within the context of acute inflammation.

### Large molecule biologics

The use of large-molecule biologics to regulate inflammation has been well-established for chronic inflammatory conditions. In these settings, the biologics act as scavengers of pro-inflammatory soluble molecules circulating in the blood or as receptor antagonists (Rider et al., 2016). Many of these biological substances come in the form of monoclonal antibodies. Unfortunately, due to their long half-life and the necessity of proper cytokine signaling in acute inflammatory conditions, the application of these therapeutics comes with a significant risk of secondary infections or reactivations (Lang et al., 2012). However, large molecules with shorter half-lives may be of some utility. This includes drugs such as anakinra, an interleukin-1 receptor antagonist that has an elimination half-life of 6 hours and has been long used in the treatment of autoimmune diseases (Bedaiwi et al., 2021). Given its relatively good safety profile, it has been used in acute inflammatory conditions. Though global results have not been promising, selectivity in patient selection based on immunological profiling may provide more encouraging results (Fisher et al., 1994; Shakoory et al., 2016).

### Small molecule inhibitors and peptides

The utility of designing drugs to interfere with protein-mediated interactions was never made clearer than during the pandemic associated with COVID-19. This resurgence of interest led to the application of small molecule compounds designed to interfere with the interactions of coronavirus with the host. Remdesivir, a nucleoside analog that interferes with the RNA-dependent RNA polymerase complex used in viral replication, was quickly approved for use in COVID-19 (Kokic et al., 2021). The small molecule Janus kinase 1/2 inhibitor, known as baricitinib, was also found to be effective with increased efficacy when combined with remdesivir in patients with COVID-19 (Kalil et al., 2021). An additional trial (NCT04311697) using synthetic vasoactive intestinal peptide (aviptadil) in the treatment of COVID-19 is currently awaiting results, overall demonstrating the feasibility and efficacy of these therapeutics in acute inflammatory diseases. Beyond this, the discovery of future small molecule compounds is likely to accelerate with the development of high-throughput compound screening libraries. For instance, utilizing such a library revealed that niclosamide, approved as an anti-helminthic drug, had broad anti-inflammatory properties via inflammasome inhibition that worked against coronavirus, as well as several different TLR ligands (de Almeida et al., 2022). Similarly, cell-penetrating peptides (CPPs), along with naturally occurring antimicrobial peptides, have been suggested to have therapeutic potential in modulating protein-mediated interactions. Several compounds have been shown to affect intracellular pathogens as well as regulate infection-mediated endothelial barrier dysfunction (Buccini et al., 2020; Koch and Stark, 2022). However, despite the pre-clinical promise of CPPs, none have been approved for clinical use thus far (Xie et al., 2020).

### MiRNA therapeutics

Manipulation of protein production and signal transduction by miRNAs has been previously discussed. Given the wide impact miRNAs can have on proteins, it makes them an attractive potential therapeutic avenue for targeting cellular processes (Rupaimoole and Slack, 2017). Data thus far regarding the therapeutic potential of miRNAs in the treatment of disease have been limited. Several clinical trials utilizing miRNAs have been performed targeting cancers or chronic viral infections (Janssen et al., 2013; Hong et al., 2020). In addition, there are several ongoing clinical trials targeting miRNAs to improve outcomes in ulcerative colitis (NCT04023396) or myocardial infarction (NCT05350969). While currently there are no miRNA therapeutics aimed at regulating acute, acquired inflammatory diseases, such as sepsis or COVID-19, the concept has gained some traction (Giza et al., 2016). Regarding COVID-19, there is currently an ongoing clinical trial examining the use of exosomes containing miRNA to treat COVID-19-mediated inflammation (NCT05216562). Though the therapeutic application of miRNAs to successfully treat acute inflammatory diseases remains to be seen, they nevertheless continue to be a topic of significant investment.

## Conclusion

Great strides have been made in recent decades that have begun to pull back the veil of complexity that is the host’s defense to acute inflammatory challenge. With the advent of more sophisticated techniques, coupled with enhanced computing tools and machine learning, we now know more about the interconnected and dynamic proteome involved in modulating a host’s response. This knowledge has been further expanded by understanding new facets of the role of the genome in producing non-coding RNA, which regulate protein structure and function, as well as the importance of multi-protein complexes forming the appropriate metabolic response to an inflammatory challenge. As these protein-mediated interactions are further elucidated, high-throughput screening techniques that repurpose old drugs, or allow for the creation of new ones, will hopefully allow for the correction of aberrant protein interactions that instigate and perpetuate acute inflammatory conditions. The development of such compounds will no doubt usher in a new era of therapeutics that will provide improved outcomes for those patients suffering from diseases associated with acute inflammation.
